# Active 2D-DNA Fingerprinting of WirelessHART Adapters to Ensure Operational Integrity in Industrial Systems

**DOI:** 10.3390/s22134906

**Published:** 2022-06-29

**Authors:** Willie H. Mims, Michael A. Temple, Robert F. Mills

**Affiliations:** Department of Electrical and Computer Engineering, US Air Force Institute of Technology, Wright-Patterson AFB, OH 45433, USA; willie.mims@afit.edu (W.H.M.); robert.mills@afit.edu (R.F.M.)

**Keywords:** device fingerprinting, counterfeit detection, IoT, IIoT, IR 4.0, multiple discriminant analysis, MDA, WirelessHART, wireless security

## Abstract

The need for reliable communications in industrial systems becomes more evident as industries strive to increase reliance on automation. This trend has sustained the adoption of WirelessHART communications as a key enabling technology and its operational integrity must be ensured. This paper focuses on demonstrating pre-deployment counterfeit detection using active 2D Distinct Native Attribute (2D-DNA) fingerprinting. Counterfeit detection is demonstrated using experimentally collected signals from eight commercial WirelessHART adapters. Adapter fingerprints are used to train 56 Multiple Discriminant Analysis (MDA) models with each representing five authentic network devices. The three non-modeled devices are introduced as counterfeits and a total of 840 individual authentic (modeled) versus counterfeit (non-modeled) ID verification assessments performed. Counterfeit detection is performed on a fingerprint-by-fingerprint basis with best case per-device Counterfeit Detection Rate (%CDR) estimates including 87.6% < %CDR < 99.9% and yielding an average cross-device %CDR ≈ 92.5%. This full-dimensional feature set performance was echoed by dimensionally reduced feature set performance that included per-device 87.0% < %CDR < 99.7% and average cross-device %CDR ≈ 91.4% using only 18-of-291 features—the demonstrated %CDR > 90% with an approximate 92% reduction in the number of fingerprint features is sufficiently promising for small-scale network applications and warrants further consideration.

## 1. Introduction

The adoption of wireless technologies is an ongoing trend that has “major implications” for achieving Internet of Things (IoT), Industrial Internet of Things (IIoT), and Fourth Industrial Revolution (IR 4.0) objectives [1]. Among the objectives is a desire to minimize or entirely eliminate human intervention through increased reliance on automation [2]. This includes using real-world virtualization in industrial system frameworks that integrate IoT/IIoT devices, computation, networking, and physical processes [3]. While the inherent efficiencies, cost savings, etc., brought about by hands-off automation, are certainly desirable, benefits are only realized if the supporting devices achieve their full life expectancy [4] and operational cyber security risks are mitigated. These risks include an increase in the number of wireless doorways for entering the operational attack surface that has expanded with the adoption and networked integration of IoT/IIoT devices [5]. This is particularly important when considering the potential for catastrophic failure in applications involving safety and the potential loss of life.

There have been decades of research and demonstration activity addressing the use of Radio Frequency Fingerprinting (RFF) to provide post-deployment operational protection of fielded electronic, electrical, and electromechanical devices. The various RF fingerprint features, extraction methods, and discrimination algorithms considered are aptly summarized by the most recent review provided in [6]. From the RF fingerprinting protection perspective, the methods addressed in [6] are collectively referred to herein as *passive fingerprinting* methods. That is, the fingerprint features are extracted from collected emissions of a component, subassembly, or device that is actively operating and performing its intended function. As evident in [6], passive fingerprinting methods are the most prolific and most appropriate for post-deployment protection within the field operation phase of a device’s technical lifespan [4].

The focus here is on *active fingerprinting* which is much less prolific and uses features extracted from stimulated responses of non-operating components, subassemblies, or devices. Active fingerprinting is most appropriate for pre-deployment protection (e.g., counterfeit detection) within the near-cradle phase of a device’s technical lifespan [4]. Among the numerous RFF citations in [6] are the earliest RF fingerprinting works [7,8,9,10] that formed the basis for a decade of subsequent activity involving passive DNA fingerprinting of wireless communication devices [4,11,12,13,14,15,16,17]. More recently, there has been a transition to active DNA fingerprinting [18,19,20,21] with work in [19] representing the first application of active DNA fingerprinting to wireless communication devices. Community interest in the first-look 1D-DNA fingerprinting work in [19] using WirelessHART devices motivated the next-step activity presented here and consideration of active 2D-DNA fingerprinting to provide performance improvement. In this case, 1D is referring to fingerprints generated from a single domain response (e.g., instantaneous time) and 2D is referring to fingerprints generated from a multi-domain response (e.g., time-frequency).

### 1.1. Operational Motivation

Wireless Highway Addressable Remote Transducer (WirelessHART) signaling continues to emerge as a key enabling technology for achieving the level of IoT/IIoT network integration required for effective operations. WirelessHART is one of the two most widely used industrial international standards, with the estimated number of deployed field devices reaching into the tens-of-millions [2]. Its proliferation is expected to continue as manufacturers produce hundreds-of-thousands of devices that are introduced into the supply chain each year [22]. The widespread adoption of WirelessHART throughout European and North American industries is attributable to several key factors. Some of these include [1,2,22]:

- Operation based on the legacy wired HART protocol—users can take full advantage of prior experience, prior training, prior tool purchases, etc.;
- Considerable reduction in deployment, installation, and maintenance cost—minimal to no additional infrastructure cabling required;
- Network architectural flexibility—expansion easily accommodated by adding connectivity to additional field devices and/or other nearby networks;
- Dramatic reduction in device commissioning times—device benchtop programing and field installation completed in hours versus days.

The demonstrations here are based on WirelessHART signaling given its widespread adoption and use to achieve IoT/IIoT and IR 4.0 objectives. The focus here is on relatively small-scale networks consisting of a gateway and five or more field devices. A five-device network configuration provides “sufficiently redundant mesh network operation” [22] while taking advantage of architectural flexibility to provide the required sub-network and cross-network interconnectivity within a “classical Industrial Wireless Sensor Network (ISWN) architecture” [2]. As with large-scale networks, care must be taken in small-scale WirelessHART networks supporting critical applications involving personnel safety. This is particularly important when considering the hundreds-of-thousands of WirelessHART devices that enter the supply chain annually [22].

As with all electronic devices (integrated circuits, circuit boards, sub-assemblies, etc.) entering the supply chain, there is concern that operational integrity may be compromised through the introduction of counterfeits. The extent of compromised device effects ranges from degraded functionality (slow, sluggish, inefficient operation) to premature failure (lifecycle termination)—both manifesting possible catastrophic results. The potential for this occurring can be minimized by employing near-cradle [4] pre-deployment protection measures to detect devices that have been intentionally modified or counterfeited before placing them into service. Near-cradle counterfeit protection is at one lifecycle extreme and includes two fundamentally different Radio Frequency (RF) methods: (1) active RF Identification (RFID) that exploits interrogated responses of onboard functionality that is intentionally embedded at the time of manufacture [23,24], and (2) active RF-DNA fingerprinting that exploits inherent response uniqueness resulting from component, device architecture, and manufacturing process differences [18,19,20,21].

### 1.2. Technical Motivation

The development and demonstration of *active DNA fingerprinting* methods in [19,21] to support pre-deployment protection was complementary to prior *passive DNA fingerprinting* works supporting post-deployment operational protection [4,11,12,14,15,16,17]. The distinction between passive and active DNA fingerprinting is not based on device operating conditions but rather the DNA generation process employed. Passive DNA fingerprinting uses features extracted from transmitted responses (wired or wireless) of an operating device that is physically connected within a system and performing its intended function—passive DNA fingerprinting is a method for providing *operational* protection during a device’s technical lifecycle [4]. Active DNA fingerprinting uses features extracted from externally stimulated device responses of an uninstalled non-operably connected device—active DNA fingerprinting is a method for providing *near-cradle* protection at the onset of a device’s technical lifecycle [4].

Active DNA fingerprinting was pursued here given prior success in microwave system state estimation [18,20] and integrated circuit discrimination [21]. These previous works demonstrated benefits for using wideband (energy spanning 100s to 1000s of MHz) stimulation signals as a means to increase source-to-device electromagnetic interaction and induce fingerprint feature variability and uniqueness. The degree of source-to-device interaction is fundamentally driven by the stimulating signal characteristics (e.g., time varying amplitude, phase, and/or frequency), the device’s electrical/electronic architecture complexity, and resultant higher-order multi-frequency intermodulation effects. The active DNA fingerprinting work in [21] introduced a wideband Stepped Frequency Modulated (SFM) stimulus signal that was subsequently adopted for WirelessHART adapter discrimination work in [19]. The active DNA work in [19] considered discrimination of the same four Siemens AW210 [25] and four Pepperl+Fuchs Bullet [26] WirelessHART adapters used for passive DNA discrimination assessments in [4,14].

The active DNA fingerprinting work in [19] used an SFM stimulus signal with one-dimensional DNA (1D-DNA) features extracted from instantaneous time domain device responses. Considering the eight available WirelessHART adapters, 8-choose-6 = 28 different 6-class Multiple Discriminant Analysis (MDA) models were developed to represent six authentic devices. The two held-out devices were introduced as counterfeits, and a total of 28 × 6 × 2 = 336 individual counterfeit device ID verification assessments performed. Collectively considering all 28 models and the 336 individual assessments, a Counterfeit Detection Rate of %CDR ≈ 92% was demonstrated. While community feedback for work in [19] was generally favorable, there was also a clear desire to improve upon the demonstrated %CDR to better support operational motivation objectives, i.e., decrease the number of counterfeit devices installed in the field and increase the pool of certified devices that may be introduced into the supply chain.

To improve %CDR, alternate DNA features were considered here using the experimentally collected WirelessHART device responses from [19]. It was observed that non-coherent phase transitions occur as the SFM source stimulus transitions between frequency steps. These transitions appear as time domain transients in the device output responses. Thus, two-dimensional DNA (2D-DNA) features are considered here using a Gabor Transform (GT), given its prior success in [13] for signals exhibiting similar transient effects, and to obtain signal information based on the local distribution of signal energy as a function of frequency. Demonstrations are performed here in light of targeting small-scale (e.g., five sensors) IoT/IIoT and IR 4.0 implementations. Relative to results in [19], demonstrations here are based on (1) a total of 8-choose-5 = 56 different 5-class MDA models—factor-of-2 increase in the number of models, with (2) three held-out counterfeit devices per model used to perform 56 × 5 × 3 = 840 individual counterfeit device ID verification assessments—factor-of-2.5 increase in the number of counterfeit assessments.

## 2. Demonstration Methodology

This section presents details for the sequence of steps taken to conduct experimental demonstrations and generate the results presented in Section 3. Summary details are provided for each step in the indicated subsection. These steps include:

- Response Collection and Processing in Section 2.1—this includes Device Under Test (DUT) emplacement, DUT stimulation, DUT stimulated output collection, and pre-fingerprint generation signal processing (filtering and decimation) to reduce computational complexity and improve discriminability;
- 1D Time Domain DNA (TdDna) Fingerprint Generation in Section 2.2—this includes generation of device TdDna fingerprints used to provide a performance baseline representing the pre-existing 1D-DNA fingerprinting capability;
- 2D Gabor Transform DNA (GtDna) Fingerprint Generation in Section 2.3—this includes generation of device GtDna fingerprints used to demonstrate performance benefits of 2D-DNA fingerprinting considered herein;
- Multiple Discriminant Analysis (MDA) in Section 2.4—this includes cross-validated training of the MDA models required for device discrimination assessments;
- Device Discrimination in Section 2.5—this includes implementation of multi-model MDA device classification as a necessary precursor to implementing the device ID verification process to perform counterfeit detection and estimate %CDR;
- Dimensional Reduction Analysis (DRA) in Section 2.5—this includes final actions taken to reduce the number of fingerprint features required to achieve a given level of discrimination performance while improving computational efficiency;

### 2.1. Response Collection and Processing

**Figure 1:**
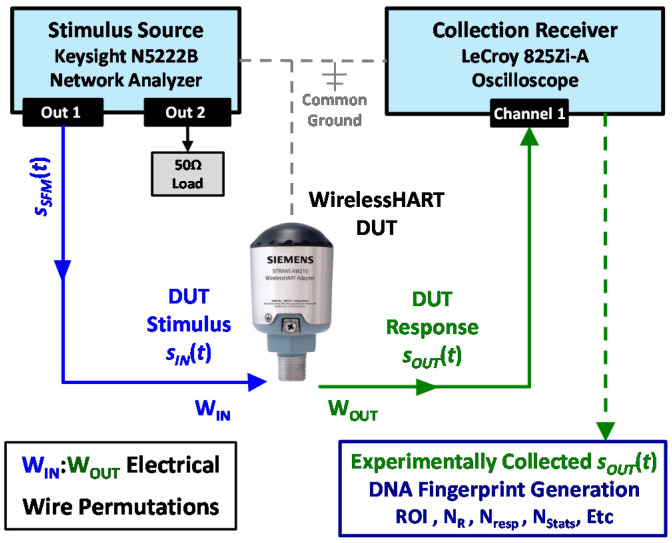
Experimental setup similar to that used in [19] for collecting WirelessHART DUT responses used for active DNA fingerprint generation.

The experimentally collected signals used here for demonstration were originally collected in support of work reported in [19] using the setup shown in Figure 1. The basic setup was adopted from integrated circuit anti-counterfeiting work originally developed and demonstrated in [21] and includes three main hardware elements: (1) a Keysight N5222B PNA microwave network analyzer [27] used to provide the DUT input stimulus $s_{\mathrm{IN}}\left(t\right)$, (2) a LeCroy WaveMaster 825Zi-A oscilloscope [28] used to collect the DUT output response $s_{\mathrm{OUT}}\left(t\right)$, and (3) a WirelessHART adapter serving as the DUT. The N5222B source parameters were set to produce the SFM stimulus signal $s_{\mathrm{SFM}}\left(t\right)$ which was power divided and input as $s_{\mathrm{IN}}\left(t\right)$ to 1-of-5 available DUT wires denoted as $W_{\mathrm{IN}}^{j}$ for *j*∈{1, 2, …, 5}.

Given a goal of maximizing the SFM source-to-DUT electromagnetic interaction and increasing DUT discrimination, the SFM stimulus parameters were empirically determined based on discrete settings available on the N5222B analyzer. The resultant SFM parameters used for experimental collection included [19] (1) a total of *N*_SFM_ = 9 frequency steps spanning a total frequency range of approximately 400 MHz < *f* < 450 MHz, with (2) the duration of each frequency step being *T*_Δ_ = 0.125 ms for a total SFM pulse duration of *T*_SFM_ = 1.125 ms. The DUT $s_{\mathrm{OUT}}\left(t\right)$ response was collected by the 825Zi-A oscilloscope, digitized, and stored for subsequent post-collection processing.

For the SFM stimulus applied to a given $W_{\mathrm{IN}}^{j}$ for *j*∈{1, 2, …, 5}, the DUT output response $s_{\mathrm{OUT}}\left(t\right)$ was collected from one of the remaining wires, denoted as $W_{\mathrm{OUT}}^{k}$ *k* ∈ {1, 2, …, 5} for *k* ≠ *j*. Given there are five connection wires on the WirelessHART adapters, there are 20 order-matters permutations of $W_{\mathrm{IN}}^{j}:W_{\mathrm{OUT}}^{k}$ (stimulus input versus output response) wire pairs that could be considered for active DNA fingerprinting. Demonstrations here are based on collections made in [19], with $W_{\mathrm{IN}}^{j}$ being the device Direct Power connecting wire and $W_{\mathrm{OUT}}^{k}$ being the device HART Signal connecting wire. It was empirically determined that this wire pair consistently yielded discernable device responses and consistent fingerprint features across all eight experimental devices.

To reduce computational complexity and improve device discrimination relative to results reported in [19], additional pre-fingerprint generation processing was introduced here. For each collected device response, the sequential pre-fingerprint generation processing included (a) BandPass (BP) filtering at the as-collected center frequency of *f*_Col_ = 425 MHz using a 16th-order Butterworth filter having a passband of W_BP_ = 50 MHz, (b) Down-Conversion (D/C) to near-BaseBand (BB) using a frequency of *f*_D/C_ = 375 MHz, (c) post-D/C BP filtering at a center frequency of *f*_Ctr_ = 50 MHz using a 16th-order Butterworth filter having a passband of W_BP_ = 50 MHz, and (d) sample decimation by a factor of five. Thus, each of the as-received WirelessHART responses at a sample rate of *f*_S_ = 1 GSps (1,115,000 samples per pulse) were converted to an *f*_S_ = 200 MSps rate (230,000 samples per pulse) prior to fingerprint generation.

**Figure 2:**
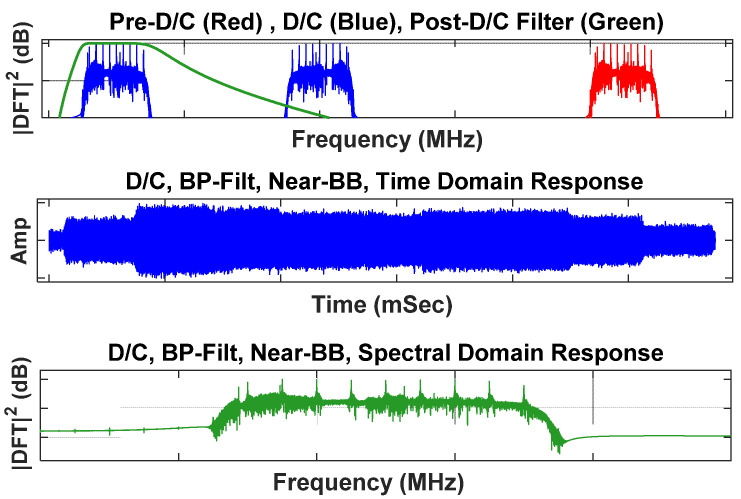
Illustration of pre-fingerprint generation processing effects on a representative WirelessHART response. The final down-converted bandpass filtered signal is used for subsequent time domain DNA (TdDna) and Gabor transform DNA (GtDna) generation.

The impact of pre-fingerprint generation processing for a representative WirelessHART response is shown in Figure 2. This processing was performed for collections from each of the *N*_Dev_ = 8 WirelessHART adapters (D1, D2, …, D8) listed in Table 1. Although the Siemens AW210 [25] and Pepperl+Fuchs Bullet [26] device labels make it appear that the devices are from two different manufacturers, it was determined that these devices are actually from the same manufacturer and were distributed under two different labels with dissimilar serial number sequencing—this difference is a result of company ownership transition to Pepperl+Fuchs. Thus, the discrimination conditions being considered represent the most challenging, like-model intra-manufacturer conditions using identical hardware devices that vary by serial number.

Table 1 shows that the devices were received with two different versions of operating firmware—the firmware version number is available when the devices are connected to the gateway. In an attempt to remove firmware as an experimental variable, the manufacturer was contacted about having the D1 and D3 devices reprogrammed with the version 200 firmware. Researchers were told that this was not a customer support option and thus firmware remained as an uncontrolled experimental variable. As supported by results presented here in Section 3 and previous related work [4,14,19] using these same devices, there is no correlation between firmware version and device discriminability.

### 2.2. 1D Time Domain DNA (TdDna) Fingerprint Generation

**Figure 3:**
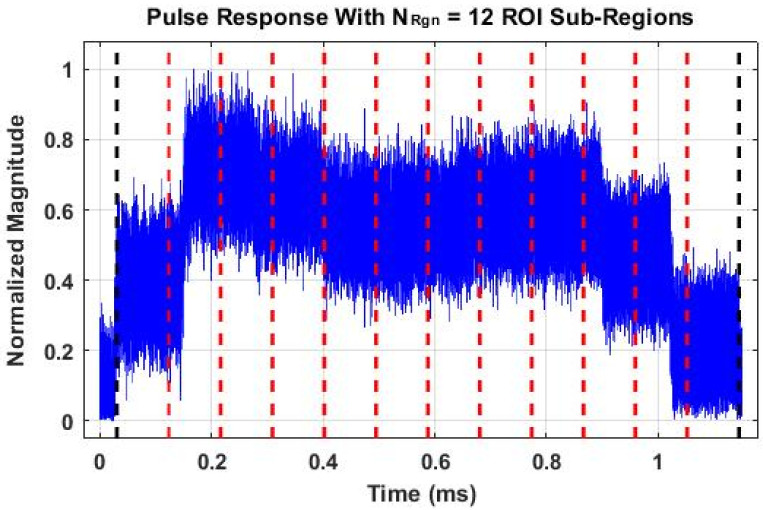
Normalized time domain magnitude of a representative DUT stimulated output showing the full ROI (bounded by black dashed lines) and the *N*_Rgn_ = 12 subregion boundaries used for generating statistical time domain DNA fingerprint features.

The time domain DNA (TdDna) fingerprint generation process used here has evolved over time and has been predominantly used in wireless passive DNA fingerprinting applications [4,13,14,15,18]. Selected process details are presented here for completeness, and the reader is referred to [4] for more details. Statistical fingerprint features are calculated from instantaneous responses of the down-converted, bandpass filtered, near-baseband pulses such as illustrated in Figure 3. Denoting the real-valued sample sequence as {s_Out_(*n*)}, the fingerprinted responses include instantaneous (1) *magnitude* calculated as M(*n*) = |s_Out_(*n*)}|, (2) *phase* calculated as Θ(*n*) = tan^−1^[H_Q_(*n*)/H_R_(*n*)] where H_R_(*n*) and H_Q_(*n*) are real and imaginary components of the Hilbert Transform [29] denoted by Hilbert[s_Out_(*n*)], and (3) *frequency* calculated as Φ(*n*) = gradient [Θ(*n*)].

A Region Of Interest (ROI) within {s_Out_(*n*)} is selected and the corresponding instantaneous {Μ(*n*)}, {Θ(*n*)} and {Φ(*n*)} response sequences are divided into *N*_Rgn_ subregions for statistical feature calculation. This is illustrated in Figure 3, which shows the {Μ(*n*)} magnitude response for the representative pulse shown in Figure 2. Considering the calculation of three statistics (variance, skewness and kurtosis [30]) using samples within each of the *N*_Rgn_ = 12 indicated subregions, and across all samples within entire ROI as well, the time domain fingerprints include a total of *N*_FD,TD_ = (12+ 1) × 3 × 3 = 117 features when accounting for the three instantaneous {Μ(*n*)}, {Θ(*n*)} and {Φ(*n*)} response sequences.

### 2.3. 2D Gabor Transform DNA (GtDna) Fingerprint Generation

Consideration of Gabor transform features is motivated by related historical work [12,13,31,32,33] that considered detection, characterization and exploitation of transient and nonlinear effects in time varying signals. These effects are manifest in the Gabor transform space as localized signal energy distributions that vary as a function of frequency. The N5222B source inherently produces an SFM pulse having a non-uniform amplitude response across time. As evident in Figure 3, this variation is generally preserved in the device output response although altered by the SFM-to-DUT interaction. Empirical analysis showed that response amplitude transitions at all step boundaries corresponded to instantaneous phase transients that randomly varied at each boundary on a pulse-by-pulse basis for all devices. Thus, device responses here possess transient characteristics consistent with those considered in [12,13,31,32], and the Gabor transform was deemed to be a reasonable first choice for 2D DNA fingerprint generation.

The *mk*^th^ Gabor transform coefficient for sampled signal *s*(*n*) is given by [12,13,31,33]

(1) $$G_{mk}=\overset{M_{T}N_{\Delta }}{\underset{n=1}{\sum }}s\left(n\right)W^{*}\left(n-mN_{\Delta }\right)e^{-j2\pi kn/M_{F}},$$

where *W*(*n*) is a given synthesis window of width *W*_τ_, * denotes complex conjugate, *m* = 1, 2, …, *M_T_*, where *M_T_* is the total number of time index shifts, *k* = 0, 1, …, *M_F_* – 1, where *M_F_* is the total number of frequency index shifts, and *N*_Δ_ is the number of samples shifted between transformations. Additional parameter constraints for the Gabor transform given by (1) include *M_F_* ≥ *N*_Δ_ and *mod*(*M_T_* × *N*_Δ_, *M_F_*) = 0. Consistent with [12,13,31], an energy normalized Hamming window was used for *W*(*n*).

**Figure 4:**
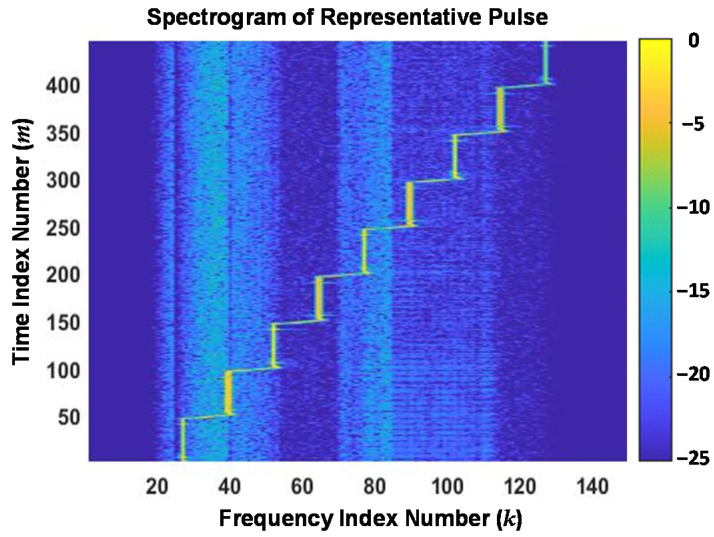
Normalized time-frequency spectrogram of the *same* representative DUT stimulated output used to generate the Figure 3 time domain magnitude response.

**Figure 5:**
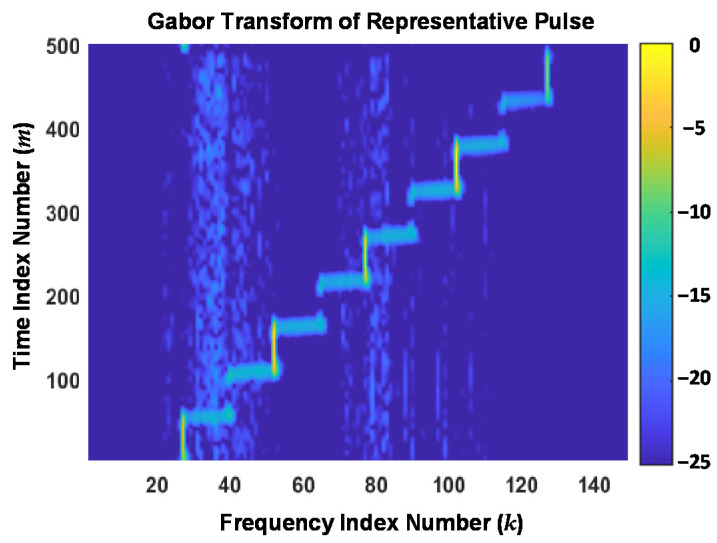
Normalized time-frequency Gabor transform response of the *same* representative DUT stimulated output used to generate the Figure 3 time domain magnitude response.

The utility of Gabor transformation for highlighting transients and localizing energy concentration is evident by comparing the conventional spectrogram response in Figure 4 with the Gabor transform response in Figure 5. These responses are shown using the same color bar scale (dB) and were generated using the representative SFM response used for Figure 3. The Gabor transform response was generated using a window width of *W_τ_* = 1 × 10^−3^, *N*_Δ_ = 460, *M_T_* = 500, and *M_F_* = 500 for a resultant (*M_T_* × *N*_Δ_)/*M_F_* = 460. As implemented here, the transformation for *M_F_* = 500 > *N*_Δ_ = 460 represents *oversampling* conditions, which are generally desirable when processing noisy data [31,33].

Relative to the spectrogram response, it is evident in Figure 5 that the highest degree of Gabor localization occurs around 3-of-9 frequency step regions (*k* = 55, 82, 109) and a moderate degree of localization occurs around extreme frequency step regions (*k* = 28, 136). The Gabor localization effects are also evident by comparing low energy time-frequency regions where the Gabor response exhibits a more distinct structure. Representative examples of this include the response regions bounded within (1) *k* ∈ {20, 21, …, 58} and *m* ∈ {175, 176, …, 500}, and (2) *k* ∈ {70, 71, …, 116} and *m* ∈ {1, 2, …, 175}.

**Figure 6:**
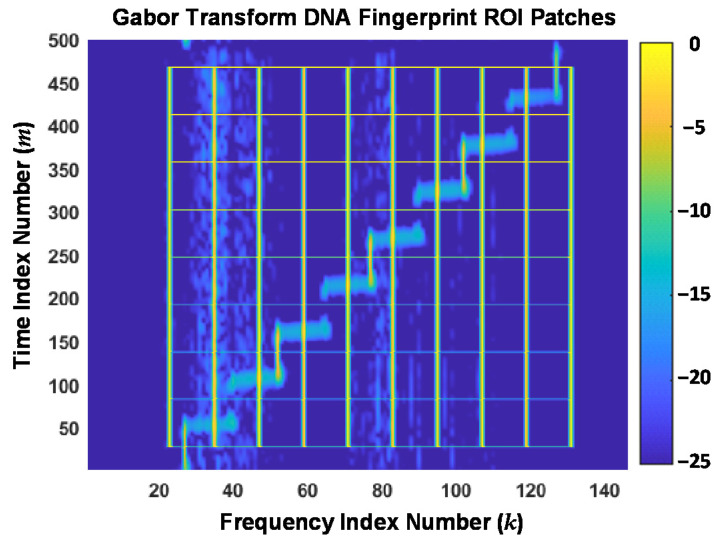
Overlay of the Gabor transform magnitude response from Figure 5 with the selected 2D fingerprinting ROI patch boundaries overlaid.

The Gabor transform features are generated from a given 2D Gabor transform response, e.g., the normalized magnitude response such as shown in Figure 5. As shown in Figure 6, a fingerprinting ROI is identified and divided into *N*_Tim_ × *N*_Frq_ 2D subregions (patches), where *N*_Tim_ and *N*_Frq_ are the number of time dimension and frequency dimension indices defining each patch. Fingerprint generation indexing is set such that the ROI is uniformly divided into *N*_TimBlcks_ and *N*_FrqBlcks_ along the time and frequency dimensions. Figure 6 shows the overlay of ROI patches used here for generating demonstration results.

The ROI overlay in Figure 6 was generated using *N*_TimBlcks_ = 8 and *N*_FrqBlcks_ = 9, with a total number of *N*_Tim_ = 55 and *N*_Frq_ = 12 indices defining the block time-frequency extent. Thus, there are a total of *N*_Ptch_ = 8 × 9 = 72 total time-frequency patches with each patch containing a total of 55 × 12 = 660 elements. Fingerprint statistics (variance, skewness, and kurtosis statistics [30]) were calculated using elements within each of the *N*_Ptch_ = 72 patches, and across all ROI elements as well, such that the resultant Gabor transform DNA fingerprints included a total of *N*_FD-GT_ = (72 + 1) × 3 = 219 statistical features.

### 2.4. Multiple Discriminant Analysis (MDA)

The MDA-based discrimination methodology used here was primarily adopted from related work in [4] that exploited passive DNA features and work in [19] that exploited passive DNA features. Both of these earlier works considered the same *N*_Dev_ = 8 WirelessHART adapters shown in Table 1 and used here for demonstration. The fundamental differences (processing, objectives, etc.) between active and passive DNA fingerprinting preclude direct comparison of results presented here with those presented in [4]. While providing a motivational basis for the active DNA fingerprinting work undertaken here, care is taken in making comparisons of results in [19] with those provided here—this is addressed further in Section 3 results. However, the MDA processing is fundamentally the same and limited details for the device discrimination process are presented here for completeness. The reader is referred to [4] for a more detailed description and development of MDA-based device classification and device ID verification.

The *N*_Dev_ = 8 WirelessHART devices in Table 1 were used to perform discrimination assessments for the *N*_Mdl_ = 56 (8-choose-5) model conditions shown in Table 2. As indicated, each model included *N*_Cls_ = 5 classes with each class represented by one of the designated Authentic (A) devices. The remaining three held-out devices were introduced as Counterfeit (C) devices for each of the modeled devices. As adopted from [4], the trained MDA model elements are denoted by (**W**, **μ_F_**, **σ_F_**, **μ***_n_*, **Σ***_n_*) where (1) **W** is the MDA projection matrix (dimension *N*_Feat_ × *N*_Cls_ − 1), (2) **μ_F_** is the input fingerprint mean normalization factor (dimension 1 × *N*_Feat_), (3) **σ_F_** is the input fingerprint standard deviation normalization factor (dimension 1 × *N*_Feat_), (4) **μ***_n_* is the projected training class means (dimension 1 × *N*_CLS_ − 1), and (5) **Σ***_n_* is the training class covariance matrix (dimension *N*_Cls_ − 1 × *N*_Cls_ − 1).

### 2.5. Device Discrimination

The trained MDA models were used for both *Device Classification* and *Device ID Verification* assessments. Given a trained (W, **μ_F_**, **σ_F_**, **μ***_n_*, **Σ***_n_*) MDA model, a fingerprint from an unknown device, denoted as **F**_Unk_ (dimension 1x*N*_Feat_), is projected into the MDA decision space using $p_{\mathrm{Unk}}=\left[\left(F_{\mathrm{Unk}}-\mu_{F}\right)⨀\sigma_{F}^{-1}\right]W$ (dimension 1 × *N*_Dev_ − 1) [4]. The resultant $p_{\mathrm{Unk}}$ is used with a given measure of similarity to generate a test statistic (Z_Unk_) that is used for making device classification and device ID verification decision. Test statistic Z_Unk_ is a real number that is used to estimate (1) which of the *N*_Cls_ modeled devices the unknown **F**_Unk_ most closely represents—the fundamental device classification process, and (2) how much the unknown **F**_Unk_ looks like fingerprints from 1-of-*N*_Cls_ specified devices—the fundamental device ID verification process used for estimating %CDR. Consistent with results in [4,19], the Z_Unk_ test statistics used here were generated from distance-based Euclidean and probability-based Multi-Variate Normal (MVN) measures of similarity. The reader is referred to [4] for a detailed description of test statistic Z_Unk_ generation and its use in making classification and ID verification decisions.

Results for the *looks-most-like* device classification process are summarized in a confusion matrix format [34]. A representative confusion matrix is shown in Table 3 for MDA classifier testing of an *N*_Cls_ = 5 model using *N*_Tst_ = 565 unknown testing fingerprints per class. The average cross-class percent correct classification (%C) is calculated as the sum of diagonal elements divided by the total number of estimates represented in the matrix (*N*_Tst_ × *N*_Cls_). The Table 3 results yield an overall %C = [2438/(565 × 5)] × 100 ≈ 86.3%, with individual per-class performances ranging from %C_Cls_ = (403/565) × 100 ≈ 71.3% for Class 5 to %C_Cls_ = (526/565) × 100 ≈ 93.1% for Class 1.

Table 3 shows that a majority of the classification error is attributable to mutual “confusion” between Class 2 (D2) and Class 5 (D5). The classification estimates in Table 3 effectively represent Monte Carlo trials and 95% Confidence Interval (CI_95%_) analysis [35] is used throughout the paper when making comparisons and drawing conclusions. The ±CI_95%_ intervals for per-class %C_Cls_ are presented in Table 3 by way of example and are based on *N*_Tst_ = 565 Monte Carlo trials.

MDA-based confusion matrix results such as presented in Table 3 were generated for all *N*_Mdl_ = 56 MDA models in Table 2. The presentation of a large number of resultant confusion matrices is avoided in the interest of brevity. As an alternative, the average *per-model* performances are presented in a %C versus Model ID format to enable comparison across models—an overall cross-model average %C is calculated and presented as well. Results are also presented for average *per-device* performance in a %C versus Device ID format to enable comparison across devices—an overall cross-device average %C is calculated and presented as well. These results are obtained by considering diagonal confusion matrix entries (correct estimates) for a given device in all models where that device is serving in an authentic role. Thus, the per-device %C averages are based on diagonal per-class entries in 35-of-56 models.

Results for the *looks-how-much like* device ID verification (counterfeit detection) process were generated using the same *N*_Mdl_ = 56 MDA models used for device classification. Counterfeit device ID verification assessments for a given model are denoted by $D_{i}^{U}:D_{k}$ (counterfeit:authentic), where $D_{i}^{U}$ is one of three non-modeled counterfeit devices and $D_{k}$ is each of the modeled authentic devices. Considering model M54 in Table 2 as an example, there are a total of 3 × 5 = 15 $D_{i}^{U}:D_{k}$ counterfeit assessments performed for all *i*∈{1, 2, 5} and all *k*∈{3, 4, 6, 7, 8}. Accounting for all *N*_Mdl_ = 56 models, there were a total of 15 × 56 = 840 individual counterfeit detection assessments completed for estimating %CDR.

The $D_{i}^{U}:D_{k}$ counterfeit ID verification assessments and %CDR estimation are based on a binary accept/reject declaration process. The accept/reject decisions are made on a fingerprint-by-fingerprint basis using the following:

- Generating a $p_{\mathrm{Unk}}^{i}=\left[\left(F_{\mathrm{Unk}}^{i}-\mu_{F}\right)⨀\sigma_{F}^{-1}\right]W$ fingerprint projection for each of the *N*_Tst_ fingerprints from the counterfeit $D_{i}^{U}$ device;
- Calculating the test statistic $Z_{\mathrm{Unk}}^{i,k}$ associated with the claimed authentic $D_{k}$ device using each of the counterfeit $p_{\mathrm{Unk}}^{i}$ projections;
- Performing a $Z_{\mathrm{Unk}}^{i,k}$ ⪋ $t_{k}^{V}$ threshold comparison, where $t_{k}^{V}$ is the device-dependent ID verification for the claimed authentic device $D_{k}$ and the ⪋ inequality condition is set as (a) greater than (>) for a higher-is-better match statistic (e.g., MVN probability), or (b) less than (<) for a lower-is-better match statistic (e.g., Euclidean Distance);
- Making a binary accept/reject declaration based on threshold criteria with (a) an accept (false positive) being an *undesirable* outcome—counterfeit not detected, and (b) a reject (true negative) being a *desirable* outcome—counterfeit detected;
- Calculating %CDR = [(*N*_Tst_ − *N*_Rej_)/*N*_Tst_] × 100 as an estimate of counterfeit detectability, where *N*_Rej_ is the total number of binary reject decisions.

As with device classification, counterfeit device ID verification results are presented in two formats. The first is a per-model %CDR versus Model ID format. In this case, the %CDR average is based on averaging binary reject decisions for three different counterfeit devices (e.g., D1, D2 and D5 in M54) being compared against all five authentic devices (e.g., D3, D4, D6, D7, D8 in M54)—the per-model %CDR averages are based on a total of 3 × 5 = 15 counterfeit ID verification assessments. The second presentation format includes per-device %CDR versus Device ID. In this case, the presented %CDR average is based on averaging reject decisions for a given device when serving as a counterfeit in 21-of-56 models (e.g., D1 in M36–M56) and being compared against all other devices—the per-device %CDR averages are based on a total of 21 × 5 = 105 counterfeit ID verification assessments.

### 2.6. Dimensional Reduction Analysis (DRA)

Processing efficiency improvement can be obtained using fingerprints having a reduced number of DRA selected (*N*_DRA_) features. The DRA features are selected as a proper subset of the Full-Dimensional (FD) feature set containing *N*_FD_ features with a goal of minimizing the impact (degradation) in classifier %C performance. DRA feature selection was performed here using classification results of (1) an Artificial Neural Network (ANN) based Learning Vector Quantization (LVQ) process adopted from [12], and (2) ensemble based Random Forest (RndF) process adopted from [14]. While there are certainly other feature selection methods that could be considered, the FD-vs-DRA classification results in Section 3 show that both methods are sufficiently robust for demonstration purposes. Unlike MDA classification, these classifiers provide a direct indication of feature relevance (importance) on the final classification decision.

Relative to MDA, there are increased computation costs with implementing ANN-based and ensemble-based classifiers. Thus, their envisioned use is limited to pre-deployment training and feature selection, with a goal of identifying DRA subsets of sufficiently relevant features that can be used with the more computationally efficient MDA-based discrimination processes described in Section 2.4. The DRA subsets are referred to herein as RndF-selected and LVQ-selected subsets and include a total of *N*_DRA_ < *N*_FD_ features. The total percentage of DRA reduction is calculated as [(*N*_FD_ − *N*_DRA_)/*N*_FD_)/] × 100.

**Figure 7:**
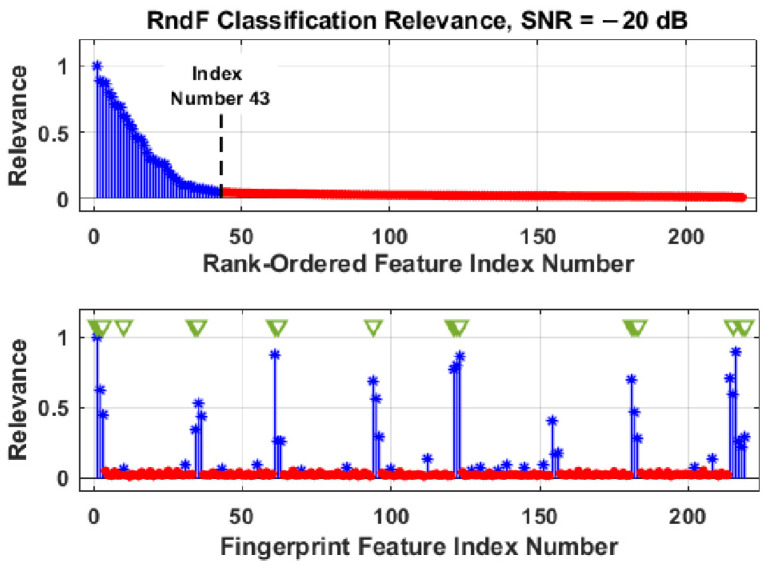
Stem plots illustrating DRA feature selection for RndF classification using the *N*_FD_ = 219 full-dimensional feature set. The blue asterisk (**∗**) markers denote the most relevant *N*_DRA,RndF_ = 43 features, the red dot (●) markers denote the least relevant 176-of-219 features, and the green triangle (**▽**) markers identify selected features shared in common with LVQ in Figure 8.

**Figure 8:**
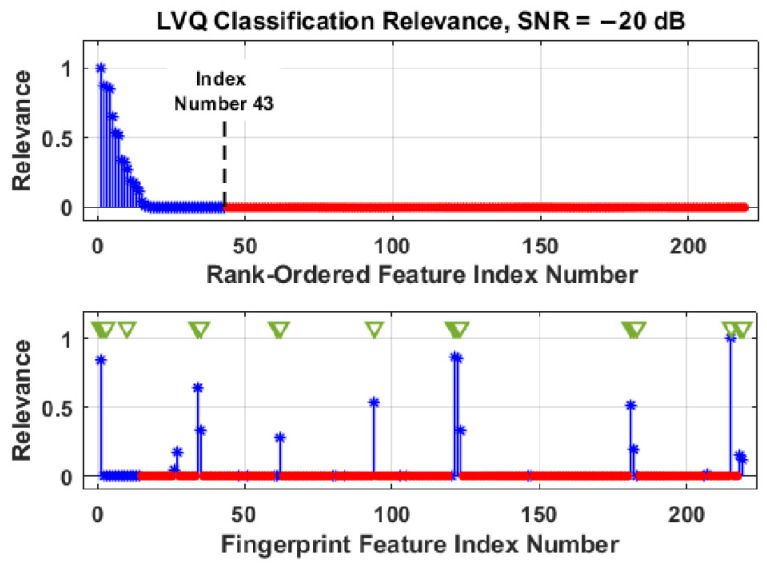
Stem plots illustrating DRA feature selection for LVQ classification using the *N*_FD_ = 219 full-dimensional feature set. The blue asterisk (**∗**) markers denote the most relevant *N*_DRA,LVQ_ = 43 features, the red dot (●) markers denote the least relevant 176-of-219 features, and the green triangle (**▽**) markers identify selected features shared in common with RndF in Figure 7.

The DRA feature selection process is illustrated in the stem plots provided in Figure 7 and Figure 8. These plots show post-classification RndF and LVQ relevance metrics for the *N*_FD_ = 219 full-dimensional feature set at SNR = −20 dB. The SNR conditions are noted given that feature relevance and DRA selection are generally SNR dependent, with a greater number of features becoming increasingly relevant as SNR decreases. The post-classification *N*_DRA_ = 43 selected features are denoted by blue asterisk (**∗**) markers and the remaining least relevant 176-of-219 features are denoted by the red dot (●) markers. The 43-of-219 selection represents an approximate 80% reduction.

The top plots in Figure 7 and Figure 8 show the sorted (highest-to-lowest relevance) rank-ordered features with a majority of the *N*_FD_ = 219 total features having zero to near-zero relevance and contributing minimally to the final classification decision. The bottom plot in each figure shows the unsorted feature relevance and the actual fingerprint feature indices for the most relevant features. The green triangle (**▽**) markers in each of the bottom plots denote 18 common features identified by both the RndF and LVQ classifiers.

The common features in Figure 7 and Figure 8 were used as an additional jointly selected feature set containing *N*_Joint_ = 18 features, i.e., the joint set was the intersection of the *N*_DRA,RndF_ = 43 RndF-selected and *N*_DRA,LVQ_ = 43 LVQ-selected feature sets. The impact of DRA on classification is determined by rerunning the classifiers and comparing the resultant %C_DRA_ using the *N*_DRA_ selected subsets with the original %C_FD_ performance. Though not the main emphasis of this section, it is interesting to note that the *N*_DRA-GT_ = 18 jointly selected feature set identified in Figure 7 and Figure 8 yielded statistically equivalent %C_FD_ and %C_DRA_ performance of %C ≈ 91% for both the RndF and LVQ classifiers—nearly a 92% reduction in the required number of features with no sacrifice in %C performance. What remains to be shown in the Results section is how the MDA classifier performs using the reduced dimensional *N*_DRA,RndF_ and *N*_DRA,LVQ_ selected feature sets.

**Figure 9:**
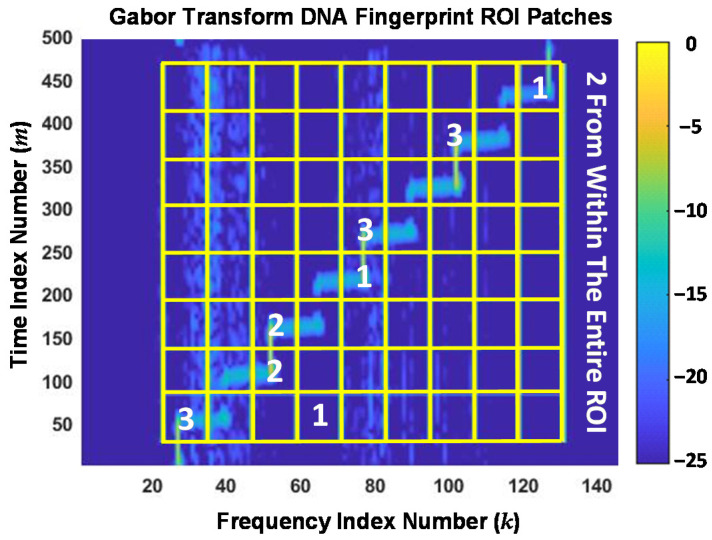
Gabor transform and ROI patch overlay from Figure 6 with numerical values added to indicate the number of jointly selected RndF and LVQ features generated within the patches. The numbers identify 1-of-18, 2-of-18, or 3-of-18 features from among the *N*_Joint_ = 18 features identified by the green triangle (**▽**) markers in Figure 7 and Figure 8.

For final discussion on DRA feature selection, it is insightful to consider where the jointly selected RndF features in Figure 7 and LVQ features in Figure 8 were generated from within the Gabor transform domain. This is illustrated in Figure 9, which shows (1) the Gabor transform response and overlaid ROI patches in Figure 6, and (2) numerical values in specific patches to indicate the number of jointly selected features generated from elements within those patches—the vertical side note indicates that 2-of-18 features were generated using all ROI elements. As indicated, all but one of the *N*_Joint_ = 18 features were generated along diagonal patches where the Gabor transform produced the maximum element-to-element energy concentration changes.

The one off-diagonal feature in Figure 9 (patch centered at *m* ≈ 50 and *k* ≈ 65) corresponds to fingerprint feature index number 10 in Figure 7 and Figure 8 and has near-zero relevance for either the RndF or LVQ classifier. A cursory analysis of device discrimination performance with fingerprint feature number 10 removed from the jointly selected *N*_Joint_ = 18 feature set proved to be inconsequential, i.e., there was no statistically significant change in either device classification or device ID verification performance.

## 3. Device Discrimination Results

Performance of MDA models representing all *N*_Cls_ = 8 devices is first considered in Section 3.1. These results are provided to (1) highlight benefits for transitioning from 1D time domain to 2D Gabor transform fingerprint features, and (2) demonstrate the effectiveness of RndF and LVQ DRA feature selection for reducing the number of fingerprint features while maintaining acceptable discrimination performance. The benefits of 2D Gabor-based fingerprints and DRA feature selection are carried over into multi-model assessments in Section 3.2. These results include *N*_Cls_ = 5 device multi-model classification and counterfeit ID verification for the *N*_Mdl_ = 56 models in Table 2.

### 3.1. 1D vs. 2D Classification Performance

**Figure 10:**
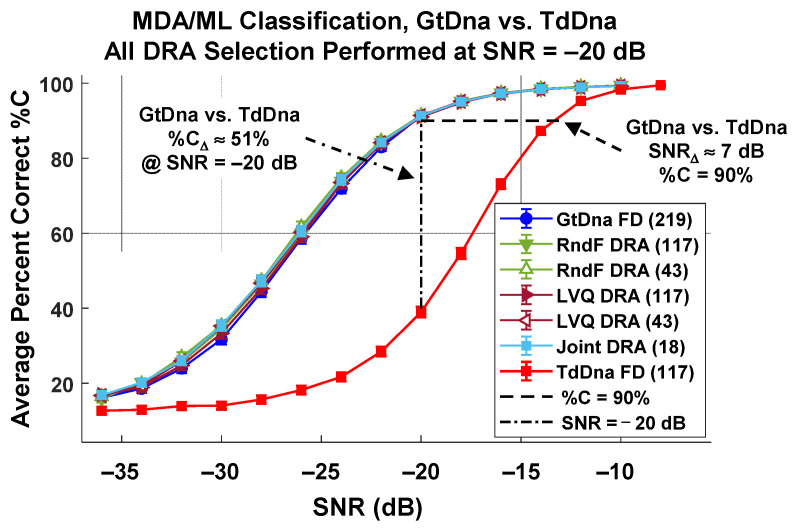
Composite MDA/ML classification performance for *N*_Cls_ = 8 devices using indicated feature sets. GtDna feature improvement is indicated by an SNR_Δ_ = SNR_GT_ − SNR_TD_ ≈ 7 dB “gain” at %C ≈ 90% and %C_Δ_ = %C_GT_ − %C_TD_ ≈ 51% improvement at SNR = −20 dB.

The first step toward characterizing discrimination performance and processing efficiency improvement included generating the full-dimensional (FD) TdDna baseline classification results for comparing with GtDna classification results. The TdDna baseline was generated using *N*_FD,TD_ = 117 TdDna features generated per Section 2.2 and the MDA/ML discrimination process in Section 2.4 for the *N*_Cls_ = 8 WirelessHART devices. The baseline TdDna FD (117) results are presented in Figure 10 and exhibit the typical trends in %C performance for variation in SNR. This includes a range of %C ≈ 100% at higher SNR representing collected response conditions and %C ≈ 1/*N*_Cls_ ≈ 12.5% at the lowest SNR representing random guessing.

The overlaid GtDna comparison results in Figure 10 were generated using the same device SFM responses. Note that each plotted data point in this figure includes the CI_95%_ intervals, and all intervals effectively span the vertical extent of the data markers. These results were generated using *N*_FD-GT_ = 219 full-dimensional and *N*_DRA_ ∈ {18, 43, 117} dimensionally reduced feature sets selected using both the RndF and LVQ relevance rank-ordering process detailed in Section 2.6. The additional “Joint” *N*_DRA_ = 18 feature set was generated as the intersection of RndF and LVQ selected feature sets. Collectively considering all plotted data points in Figure 10 and their corresponding CI_95%_ intervals, GtDna fingerprinting is superior for all but one of the SNR conditions. The one exception occurs under the highest SNR = −6 dB condition, where TdDna and GtDna performances are statistically equivalent for all fingerprint sets considered. Most notably, GtDna DRA performance is statistically equivalent to GtDna FD performance for all DRA subsets under SNR ≥ −20 dB conditions. Benefits for using the *N*_DRA_ = 18 feature set include (1) a GtDna vs. TdDna performance gain of %C_Δ_ = %C_GT_ − %C_TD_ ≈ 51%, and (2) an approximate 92% reduction in the number of required GtDna features (18 vs. 291) with no trade-off penalty incurred in average cross-class %C performance.

### 3.2. Multi-Model Discrimination

The benefits of 2D GtDna fingerprinting highlighted in Section 3.1 for the *N*_Cls_ = 8 device models were likewise observed for all *N*_Mdl_ = 56 models in Table 2. That is, statistically equivalent discrimination was achieved using the *N*_FD-GT_ = 219 and *N*_DRA-GT_ = 18 feature sets. Thus, representative results for SNR = −20 dB conditions are presented and provide the basis for detailed discussion of multi-model classification and counterfeit ID verification performance. As noted in Section 2.5, both per-model and per-device %C classification and %CDR counterfeit ID verification performances are presented.

**Figure 11:**
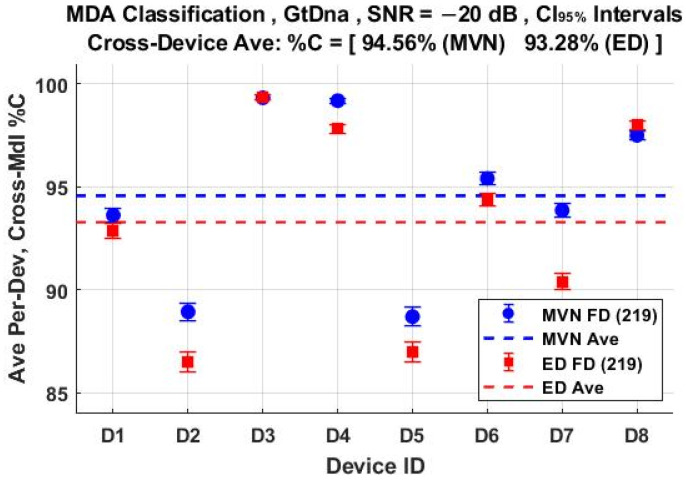
Per-device MDA classification performance for the *N*_Mdl_ = 56 models in Table 2. Results are for the full-dimensional *N*_FD-GT_ = 219 feature set generated under SNR = −20 dB conditions using ED (■) and MVN (●) measures of similarity.

**Figure 12:**
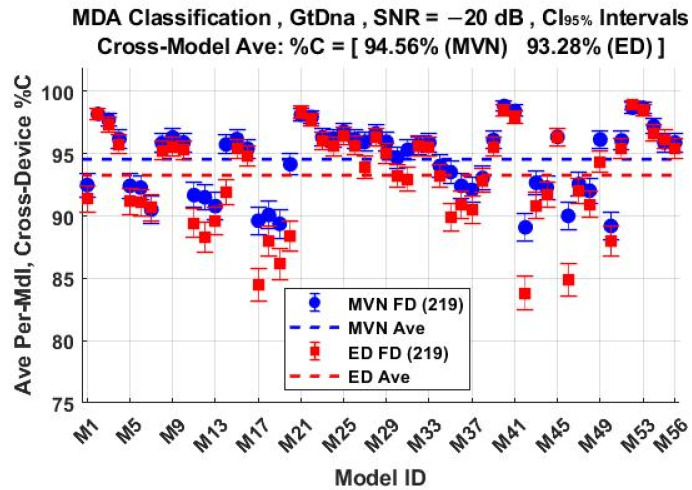
Per-model MDA classification performance for the *N*_Mdl_ = 56 models in Table 2. Results are for full-dimensional *N*_FD-GT_ = 219 features generated under SNR = −20 dB conditions using ED (■) and MVN (●) measures of similarity.

**Figure 13:**
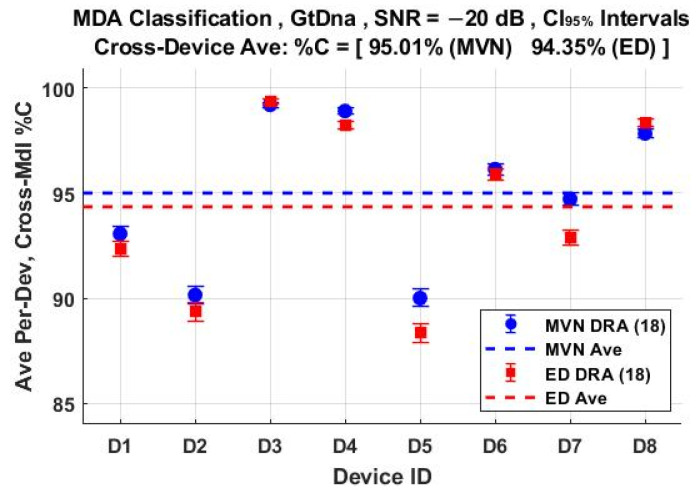
Per-device MDA classification performance for the *N*_Mdl_ = 56 models in Table 2. Results are for jointly selected *N*_DRA-GT_ = 18 features generated under SNR = −20 dB conditions using ED (■) and MVN (●) measures of similarity.

**Figure 14:**
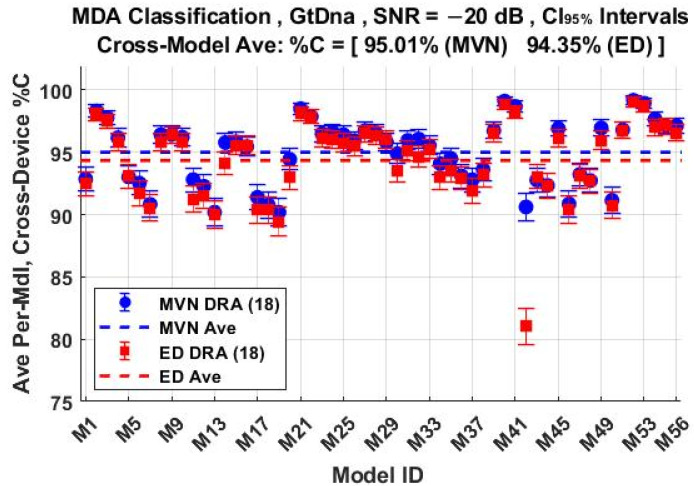
Per-model MDA classification performance for the *N*_Mdl_ = 56 models in Table 2. Results are for jointly selected *N*_DRA-GT_ = 18 features generated under SNR = −20 dB conditions using ED (■) and MVN (●) measures of similarity.

The per-device and per-model %C classification performances using the *N*_FD-GT_ = 219 feature set are shown in Figure 11 and Figure 12, respectively. These include overlaid results for both ED (■) and MVN (●) measures of similarity. The corresponding per-device and per-model %C performances for the *N*_DRA-GT_ = 18 feature set are shown in Figure 13 and Figure 14, respectively. Comparison of Figure 11 through Figure 14 shows that (1) the individual per-device and per-model results for MVN are statistically equivalent to, or better than, ED results for a majority of the individual assessments, with (2) the corresponding cross-device and cross-model averages (dashed lines) indicating that MVN provides overall marginally better performance (0.66% < %C_Δ_ = %C_MVN_ − %C_ED_ < 1.28%). The better MVN versus ED performance here is consistent with findings in [4,19].

**Figure 15:**
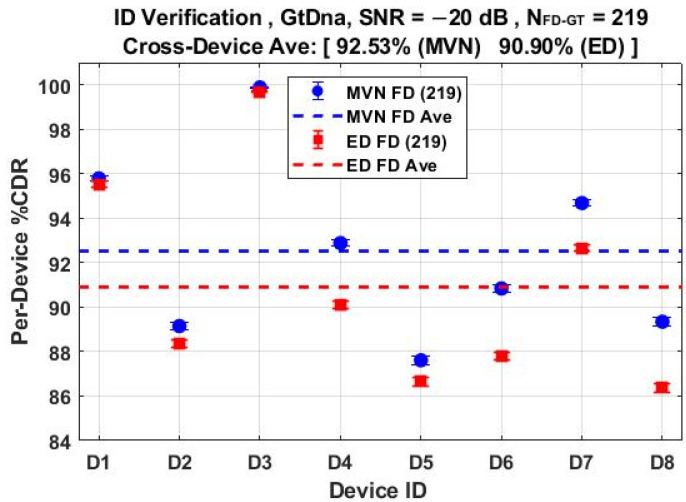
Per-device ID verification performance for full-dimensional *N*_FD-GT_ = 219 features using the *N*_Mdl_ = 56 MDA models used for generating %C results in Figure 11. Results generated under SNR = −20 dB conditions using ED (■) and MVN (●) measures of similarity.

**Figure 16:**
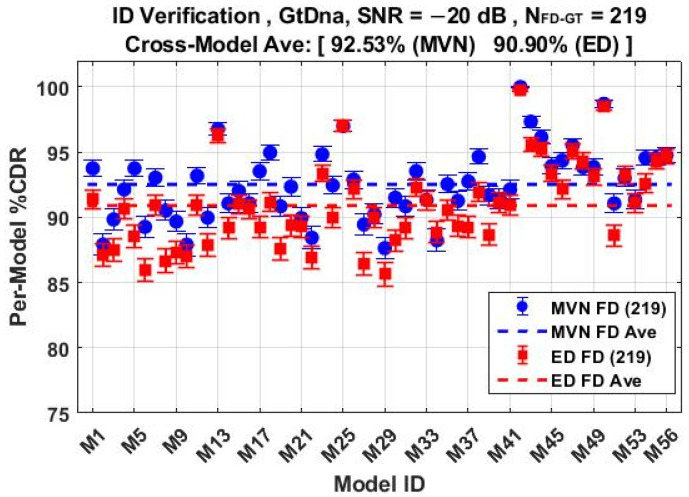
Per-model ID verification performance for full-dimensional *N*_FD-GT_ = 219 features using the *N*_Mdl_ = 56 MDA models used for generating %C results in Figure 12. Results generated under SNR = −20 dB conditions using ED (■) and MVN (●) measures of similarity.

**Figure 17:**
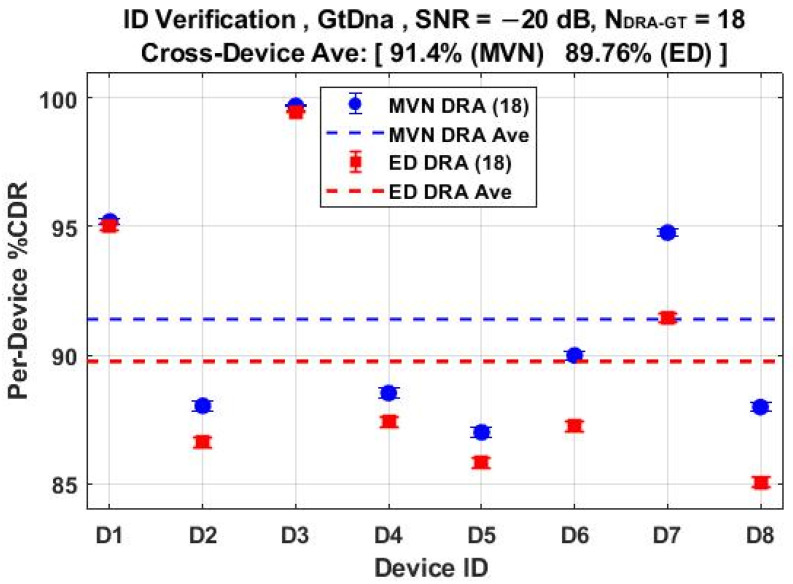
Per-device ID verification performance for jointly selected *N*_DRA-GT_ = 18 features using the *N*_Mdl_ = 56 MDA models used for generating %C results in Figure 13. Results generated under SNR = −20 dB conditions using ED (■) and MVN (●) measures of similarity.

**Figure 18:**
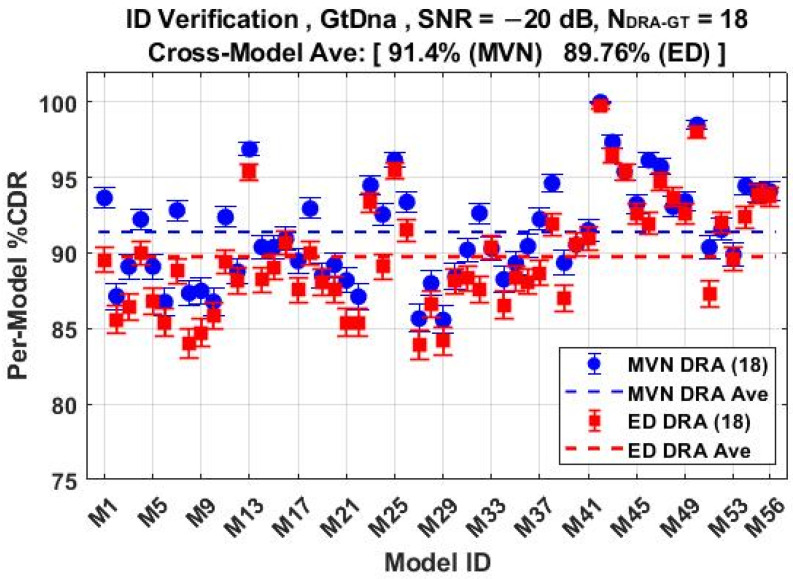
Per-model ID verification performance for jointly selected *N*_DRA-GT_ = 18 features using the *N*_Mdl_ = 56 MDA models used for generating %C results in Figure 14. Results generated under SNR = −20 dB conditions using ED (■) and MVN (●) measures of similarity.

The *N*_Mdl_ = 56 MDA models used for classification results in Figure 11 through Figure 14 were next used for counterfeit device ID verification assessments. The per-device and per-model performances for the *N*_FD-GT_ = 219 feature set are shown in Figure 15 and Figure 16, respectively, with overlaid results for both the ED (■) and MVN (●) measures. The corresponding per-device and per-model counterfeit ID verification performances for the *N*_DRA-GT_ = 18 feature set are shown in Figure 17 and Figure 18, respectively. These results collectively embody performance of 56 × 5 × 3 = 840 individual counterfeit device ID verification assessments. In comparing Figure 15 through Figure 18 results, it is evident that (1) the per-device and per-model MVN results are statistically equivalent to, or better than, ED results for a majority of the individual assessments, (2) the cross-device and cross-model %CDR averages (dashed lines) show that the MVN measure is once again marginally superior (%CDR_Δ_ = %CDR_MVN_ − %CDR_ED_ ≈ 1.6%), and (3) the per-device performance for the more efficient *N*_DRA-GT_ = 18 feature set includes 87.0% < %CDR < 99.7% and an overall cross-device average of %CDR ≈ 91.4%. This represents a 92% reduction in the required number of fingerprint features with no trade-off penalty incurred in average %CDR performance.

## 4. Summary

This work was motivated by the need for providing reliable communications in IoT/IIoT and IR 4.0 systems that are becoming increasingly reliant on automation. WirelessHART communications is one of the key technologies for achieving desired automation objectives and its operational integrity must be ensured. This is addressed using stimulated responses from eight commercial WirelessHART adapters and active 2D Distinct Native Attribute (2D-DNA) fingerprinting. The 2D-DNA fingerprints are generated from Gabor transformed responses and used to train 56 Multiple Discriminant Analysis (MDA) models. Each 5-class model represents five authentic network devices and the three non-modeled devices are introduced as counterfeits to complete 5 × 3 × 56 = 840 individual authentic (modeled) versus counterfeit (non-modeled) ID verification assessments.

Counterfeit Detection Rate (%CDR) is estimated using an MDA-based ID verification process and is the primary metric for characterizing counterfeit detectability. ID verification is performed for the 840 authentic:counterfeit assessments using binary accept/reject threshold testing. The desired outcome is a reject decision (true negative) when a counterfeit device is presented for ID verification. Relative to motivational work in [19], the statistical significance of estimated %CDR is increased here given (1) a factor-of-2 increase in the number of MDA models considered (56 vs. 28), and (2) a factor-of-2.5 increase in the number of counterfeit detection assessments performed (840 vs. 336). Processing efficiency improvement is also achieved using Dimensional Reduction Analysis (DRA) to perform feature down-selection with Random Forest (RndF) [14] and Learning Vector Quantization (LVQ) [12] classifiers. The jointly selected DRA feature set contains only 18-of-291 full-dimensional features (an approximate 92% reduction) and is an important step toward achieving computational efficiency objectives.

The %CDR estimates are based on fingerprint-by-fingerprint ID verification assessments with the best case per-device %CDR of 87.6% < %CDR_FD_ < 99.9% in Figure 15 obtained using the probability-based MVN measure of similarity. Considering all eight devices, this corresponds to average cross-device %CDR_FD_ ≈ 92.5% for the full-dimensional fingerprints. This performance was echoed by the *N*_Joint_ = 18 DRA performance in Figure 17 that includes per-device 87.0% < %CDR_DRA_ < 99.7% and average cross-device %CDR_DRA_ ≈ 91.4% using only 18-of-291 features. This represents a marginal sacrifice in %CDR performance (%CDR_FD_ − %CDR_DRA_ ≈ 1.1%) with considerable reduction in the number of required fingerprint features (18 vs. 291) and a corresponding boost in computational efficiency.

## 5. Conclusions

The %CDR > 90% here under small-scale network constraints are believed to be sufficient for motivating supply chain participants (manufacturers, distributors, customers) to consider using active 2D-DNA fingerprint features to certify field kits containing “matched” devices. The certification process could include generation of digital 2D-DNA credentials (e.g., model parameters, device fingerprint features, etc.) for certified package contents that are passed point-to-point as the package traverses the supply chain. The envisioned kit-based protection would require access to active 2D-DNA processing capability (stimulus generator, response collector, fingerprint generator, classifier) at each ID verification check point. The required processing would ideally be hosted in a relatively low cost, small form factor unit such as a Software Defined Radio (SDR) hosting an analog source generator and sufficient Field Programmable Gate Array (FPGA) processing capability to perform required post-collection fingerprint generation and discrimination.

Obtaining computational efficiency amidst limited resource constraints is a common challenge when bringing an experimental method to fruition for operational deployment. Of particular relevance to enhancing the experimental-to-operational transition potential of active 2D-DNA fingerprinting is that the %CDR > 90% here was achieved using 92% fewer fingerprint features (18-of-291) relative to what was used in prior motivational work [19]—this effectively reduces the required fingerprint generation, storage, transfer, and computation requirements. Processing improvements such as this are important, and work continues to further enhance computational efficiency. As a next step, work is underway to address digitization requirements for SDR-FPGA implementation. This specifically includes considering 2D surface quantization that must occur prior to fingerprint generation and the effect of bit depth on device discrimination.

## Figures and Tables

**Table 1:** Selected details for *N*_Dev_ = 8 WirelessHART adapters used for demonstration.

Device ID	Device Label	Firmware	Serial Number
D1	Siemens AW210	198	003095
D2	Siemens AW210	200	003159
D3	Siemens AW210	198	003097
D4	Siemens AW210	200	003150
D5	Pepperl+Fuchs Bullet	200	1A32DA
D6	Pepperl+Fuchs Bullet	200	1A32B3
D7	Pepperl+Fuchs Bullet	200	1A3226
D7	Pepperl+Fuchs Bullet	200	1A32A4

**Table 2:** Device assignments for the *N*_Mdl_ = 56 models the *N*_Dev_ = 8 adapters serving in the indicated Authentic (A) or Counterfeit (C) roles.

Model ID	D1	D2	D3	D4	D5	D6	D7	D8
M1	A	A	A	A	A	C	C	C
M2	A	A	A	A	C	A	C	C
M3	A	A	A	A	C	C	A	C
⁝	⁝	⁝	⁝	⁝	⁝	⁝	⁝	⁝
M54	C	C	A	A	C	A	A	A
M55	C	C	A	C	A	A	A	A
M56	C	C	C	A	A	A	A	A

**Table 3:** Classification confusion matrix for model M1 (D1, D2, D3, D4, D5) in Table 2 showing *N*_Cls_ = 5 discrimination performance for SNR = −20 dB conditions.

	Called Class				
Input Class	Class 1	Class 2	Class 3	Class 4	Class 5
**Class 1**	**526**	0	10	29	0
**Class 2**	0	**438**	27	0	100
**Class 3**	5	17	**539**	0	4
**Class 4**	32	0	1	**532**	0
**Class 5**	0	152	10	0	**403**
**%C_Cls_**	93.1%	77.5%	95.4%	94.2%	71.3%
**±CI_95%_**	2.1%	3.4%	1.7%	1.9%	3.7%

## Data Availability

The experimentally collected WirelessHART data supporting the reported results were not approved for public release at the time of paper submission. Requests for release of these data to a third party should be directed to the corresponding author. Data distribution to a third party will be made on a request-by-request basis and are subject to public affairs approval.

## Abbreviations

The following abbreviations are used throughout the manuscript:

%C	Average Cross-Class Percent Correct Classification
ANN	Artificial Neural Network
%CDR	Counterfeit Detection Rate Percentage
CI_95%_	95% Confidence Interval
ED	Euclidean Distance
FD	Full Dimensional
DNA	Distinct Native Attribute
DRA	Dimensional Reduction Analysis
FPGA	Field Programmable Gate Array
GT	Gabor Transform
GtDna	Gabor Transform DNA
GSps	Giga-Samples Per Second
ID	Identity/Identification
IoT	Internet of Things
IIoT	Industrial Internet of Things
IR 4.0	Industrial Revolution 4.0
LVQ	Learning Vector Quantized
MDA	Multiple Discriminant Analysis
MHz	Megahertz
MSps	Mega-Samples Per Second
MVN	Multivariate Normal
RFID	Radio Frequency Identification
RndF	Random Forest
SDR	Software Defined Radio
SFM	Stepped Frequency Modulated
TD	Time Domain
TdDna	Time Domain DNA
HART	Highway Addressable Remote Transducer
